# Vitamin D Status in People Living with HIV: Assessment of 25(OH)D Levels and Associated Factors—A Cross-Sectional Study

**DOI:** 10.3390/metabo16010083

**Published:** 2026-01-21

**Authors:** Florentina Dumitrescu, Eugenia-Andreea Marcu, Vlad Pădureanu, Livia Dragonu, Ilona-Andreea Georgescu, Lucian Giubelan, Rodica Pădureanu, Sineta Cristina Firulescu

**Affiliations:** 1 Department of Infectious Disease, University of Medicine and Pharmacy of Craiova, 200349 Craiova, Romania; florentina.dumitrescu@umfcv.ro (F.D.); livia.dragonu@umfcv.ro (L.D.); georgescuilonaa@gmail.com (I.-A.G.); lucian.giubelan@umfcv.ro (L.G.); 2 ”Victor Babes“, Hospital of Infectious Diseases and Pulmonology from Craiova, 200515 Craiova, Romania; 3 Department of Internal Medicine, Faculty of Medicine, University of Medicine and Pharmacy of Craiova, 200349 Craiova, Romania; rodica.padureanu@umfcv.ro; 4 Department of Rheumatology, Faculty of Medicine, University of Medicine and Pharmacy of Craiova, 200349 Craiova, Romania; sineta.firulescu@gmail.com

**Keywords:** HIV, vitamin D, deficiency, antiretroviral

## Abstract

Background: Vitamin D deficiency (VDD) is highly prevalent among people living with human immunodeficiency virus (HIV), with reported rates of insufficiency and deficiency substantially higher than in many general-population cohorts. This study aims to assess the prevalence of vitamin D deficiency and to investigate the risk factors contributing to its occurrence among people living with HIV who are receiving antiretroviral therapy (ART) and are registered at the Craiova Regional Center (CRC). Methods: A retrospective study was conducted from May 2024 to August 2024, including individuals with HIV aged 18 years and older who were registered at the CRC. Results: A total of 138 patients were included in the study. The prevalence of vitamin D deficiency (<20 ng/mL) and vitamin D insufficiency (20–29.9 ng/mL) was 36.2% and 33.3%, respectively, with an average vitamin D level of 26.4 ± 9.9 ng/mL. Vitamin D deficiency was associated with obesity (*p* = 0.0013), high HIV viral load (*p* = 0.043), low CD4 nadir (<200 cells/mm^3^, *p* = 0.006), prolonged ART exposure (*p* = 0.002), and the use of tenofovir disoproxil fumarate or protease inhibitor-containing regimens (*p* = 0.034 and *p* = 0.016, respectively). Conclusions: These findings indicate that monitoring vitamin D levels could be particularly relevant for patients with HIV with higher-risk profiles. However, our study included a relatively small number of participants, so further research in larger cohorts is needed to better understand these patterns.

## 1. Introduction

Vitamin D is a fat-soluble compound derived from the cholesterol precursor 7-dehydrocholesterol and belongs to the secosteroid group of molecules. The two main forms relevant to humans are vitamin D2 (ergocalciferol), which originates from plant ergosterol, and vitamin D3 (cholecalciferol), synthesized from cholesterol in animals. Dietary intake, fortified products, and supplements can provide vitamin D; however, the predominant source for the human body is its endogenous synthesis in the skin under ultraviolet radiation [1].

25-hydroxyvitamin D [25(OH)D] is the main circulating form and the most reliable marker of vitamin D status, though it is biologically inactive. The active metabolite, 1,25-dihydroxyvitamin D [1,25(OH)_2_D], regulates calcium and phosphate homeostasis and exerts autocrine and paracrine effects on immune cells. Understanding these distinct roles is essential to interpret alterations in vitamin D status among people living with HIV [2,3,4].

Vitamin D is a secosteroid hormone whose biological activity depends on a tightly regulated process of binding, transport, and enzymatic activation. After synthesis in the skin or dietary intake, vitamin D is hydroxylated in the liver to form 25-hydroxyvitamin D. In the circulation, 85–90% of 25(OH)D is bound to vitamin D-binding protein (DBP), with a smaller fraction bound to albumin and less than 1% remaining free and biologically active. The DBP–25(OH)D complex maintains bioavailability, regulates tissue delivery, and protects vitamin D metabolites from rapid degradation [3,5,6].

The biologically active form, 1,25-dihydroxyvitamin D is generated primarily in the kidneys via 1α-hydroxylation mediated by CYP27B1. Extra-renal activation also occurs in immune cells such as macrophages and dendritic cells, enabling local autocrine and paracrine immunomodulatory effects [7]. This activation pathway is tightly regulated by inflammatory signals, calcium–phosphate balance, and hormonal factors. Disruption of any step in vitamin D binding, transport, or activation—through altered DBP levels, enzymatic dysregulation, or inflammatory-mediated catabolism—can reduce the bioavailability of active vitamin D and impair immunological function [2,4].

Vitamin D deficiency (VDD) is highly prevalent among people living with human immunodeficiency virus (HIV), with observational studies and meta-analyses reporting significantly higher rates of insufficiency and deficiency compared with many general-population cohorts. Several large cohorts of adults with HIV have documented high prevalence of low serum 25(OH)D, and meta-analytic data indicate that subjects with HIV are more prone to VDD than non-HIV populations [8,9,10].

Prevalence estimates vary by study and by the cutoff used 25(OH)D, but most cohorts report a large burden of suboptimal vitamin D status that relates to traditional risk factors (reduced sun exposure, darker skin pigmentation, older age) as well as HIV-specific variables [11].

Several mechanisms have been proposed to explain altered vitamin D metabolism in the setting of HIV. Chronic inflammation and immune activation can affect vitamin D binding and activation pathways, while antiretroviral therapy (ART) exerts drug-specific effects on vitamin D status through cytochrome P450 induction or inhibition. In particular, non-nucleoside reverse transcriptase inhibitors (NNRTIs) such as efavirenz have been repeatedly associated with early and clinically meaningful declines in serum 25(OH)D after ART initiation, likely via induction of vitamin D-metabolizing cytochromes. These pharmacologic interactions complicate the interpretation of vitamin D status in patients living with HIV (PLWH) and may contribute to the high prevalence observed across cohorts [12].

Tenofovir disoproxil fumarate (TDF), a widely used nucleoside reverse transcriptase inhibitor (NRTI), has been associated with alterations in bone mineral density and vitamin D metabolism. TDF can lead to renal phosphate wasting and subtle tubulopathy, which may reduce the availability of phosphate necessary for vitamin D activation and bone mineralization. Consequently, long-term TDF exposure has been linked to lower serum 25(OH)D levels and an increased risk of osteopenia or osteoporosis in patients with HIV. These effects underscore the importance of monitoring vitamin D status in individuals receiving TDF-containing regimens [13].

Protease inhibitors (PIs) may also influence vitamin D metabolic pathways. Although clinical findings are inconsistent, PIs have been implicated in HIV-associated bone loss and may affect vitamin D metabolism through modulation of cytochrome P450 enzyme activity, which regulates hydroxylation and activation of vitamin D metabolites. These effects provide a biologically plausible mechanism linking long-term PI use to suboptimal vitamin D status and related bone outcomes [14,15].

Beyond the direct effects of ART, chronic inflammation and persistent immune activation—hallmarks of HIV infection even in patients with sustained virologic suppression—play a crucial role in disrupting vitamin D homeostasis. Ongoing immune activation is associated with elevated levels of pro-inflammatory cytokines, which may alter DBP concentrations, impair the transport of circulating 25-hydroxyvitamin D, and dysregulate the activity of key enzymes involved in vitamin D activation and catabolism. Inflammatory signaling has also been shown to upregulate vitamin D-metabolizing pathways, potentially accelerating the degradation of 25(OH)D and contributing to the high prevalence of VDD observed in PLWH. These mechanisms highlight the complex interplay between immune dysregulation, chronic inflammation, and vitamin D metabolism in HIV infection and provide a biological rationale for the associations observed between VDD, immune status, and disease progression [4,16].

Bone health

Clinical consequences of low vitamin D in HIV include adverse effects on skeletal health. Initiation of ART is associated with early bone mineral density loss, and vitamin D insufficiency likely exacerbates antiretroviral-associated bone demineralization. Several randomized and observational studies suggest that combined vitamin D and calcium supplementation can attenuate bone loss in some contexts, although effect sizes and optimal regimens vary [11,17,18]. Evidence from network meta-analyses indicates that maintaining adequate vitamin D levels may support bone health outcomes in people living with HIV, particularly in those receiving long-term ART or tenofovir-containing regimens [17].

Immune outcomes

Vitamin D also plays a role in immune modulation. Associations have been observed between low 25(OH)D levels and increased susceptibility to opportunistic infections such as tuberculosis. Mechanistic studies support a role for vitamin D in inducing antimicrobial peptides, modulating T-cell function, and regulating inflammatory cytokines [17,19]. However, causal links between vitamin D correction and improved HIV-related clinical outcomes remain incompletely established, and intervention trials show mixed results depending on baseline vitamin D status, supplementation dose, and ART regimens.

Intervention trials of vitamin D supplementation in patients living with HIV have shown that cholecalciferol reliably raises serum 25(OH)D concentrations, but evidence of benefit for non-skeletal endpoints is mixed. Some trials report improvements in surrogate immune markers or partial mitigation of ART-associated bone loss, while others show minimal clinical effect beyond correction of the biochemical deficiency. Heterogeneity in baseline vitamin D status, supplementation dose and schedule, concomitant antiretroviral regimens, and outcome measures limits cross-trial comparison and prevents definitive treatment recommendations for many HIV-specific endpoints [12,20].

Given current evidence, pragmatic clinical practice for patients with HIV commonly includes baseline assessment of vitamin D status in patients with risk factors (or when starting ART known to affect vitamin D metabolism), correction of deficiency using established repletion regimens, and consideration of maintenance supplementation tailored to ongoing risk and ART regimen. Important gaps remain: large, well-powered randomized trials are needed to determine whether routine correction of vitamin D deficiency modifies clinically important outcomes in HIV (e.g., fracture risk, tuberculosis incidence, immune recovery) and to define optimal dosing strategies across ART classes [12,13].

This study aims to assess the prevalence of vitamin D deficiency and to investigate the risk factors contributing to its occurrence among PLWH who are receiving ART and are registered at the Craiova Regional Center (CRC). According to currently available evidence, this is the first study evaluating vitamin D deficiency and its associated factors among people living with HIV in the Oltenia region of Romania, an underrepresented geographical area.

## 2. Materials and Methods

We conducted a retrospective, cross-sectional study over a duration of 4 months (May–August 2024), including individuals with HIV aged 18 years and older who were registered at the CRC. A total of 138 patients were enrolled, representing 44% of all individuals with HIV in Dolj County under CRC follow-up.

**Inclusion criteria**
  - PLWH who were registered in our clinic with complete clinical and laboratory records.
  - Patients receiving ART for at least 1 year.
**Exclusion criteria**
  - Patients with incomplete medical records.
  - Patients with coexisting conditions affecting vitamin D metabolism (such as chronic kidney disease, liver failure, or parathyroid disorders).
  - Patients receiving vitamin D supplementation prior to data collection.

For each participant, demographic and lifestyle information was collected, including gender, age, smoking status, and alcohol consumption. Clinical and laboratory data included body mass index (BMI), serum [25(OH)D] levels, CD4 cell counts, and HIV viral load (VL-HIV). Detailed records of ART regimens were obtained. ART was initiated according to national and international guidelines applicable at the time of treatment initiation, which are regularly updated [21].

CD4^+^ T-cell counts were determined by flow cytometry using the BD FACS Via system (Becton Dickinson and Company, San Jose, CA, USA), according to the manufacturer’s instructions and VL-HIV was measured by quantitative polymerase chain reaction (PCR) for HIV RNA using the Abbott m2000 system (Abbott Molecular Inc., Des Plaines, IL, USA).

[25(OH)D] levels were measured using a chemiluminescence immunoassay on the Alinity i system (Abbott Laboratories, Abbott Park, IL, USA), following the manufacturer’s instructions. Vitamin D deficiency was defined as serum 25(OH)D levels below 20 ng/mL, while vitamin D insufficiency was defined as levels between 20 and 29.9 ng/mL.

Height and body weight were measured by nurses during routine clinical assessments. BMI was then calculated for each participant using the standard formula weight (kg)/height^2^ (m^2^) and categorized according to World Health Organization (WHO) guidelines: a BMI below 18.5 kg/m^2^ was considered underweight, 18.5–24.9 kg/m^2^ as normal weight, 25–29.9 kg/m^2^ as overweight, and 30 kg/m^2^ or higher as obesity [22].

Data were obtained from the medical records and electronic database of patients registered at the CRC. Statistical analyses were performed using Microsoft Excel 2019 (Analysis ToolPak) and SPSS version 28. Continuous variables were tested for normality using the Shapiro–Wilk test. Continuous variables were analyzed using Student’s *t*-test to compare groups, and categorical variables using Chi-square or Fisher’s exact tests. A two-tailed *p*-value < 0.05 was considered significant.

To identify factors independently associated with vitamin D deficiency, a multivariate logistic regression analysis was conducted. Vitamin D deficiency was treated as a binary dependent variable (deficiency: yes/no).

Variables included in the multivariate model were selected based on clinical relevance and univariate analysis results and included: BMI (normal weight vs. overweight/obese), place of residence (rural vs. urban), CD4^+^ T-cell nadir (<200 vs. ≥200 cells/mm^3^), HIV viral load (>10.000 vs. ≤10.000 copies/mL), duration of ART (≤15 vs. >15 years), exposure to TDF, and exposure to PIs.

Adjusted odds ratios (ORs) and 95% confidence intervals (CIs) were calculated. A two-tailed *p*-value < 0.05 was considered statistically significant.

Results were presented in charts and tables. All participants provided written informed consent for the use of their medical data.

This study was conducted in accordance with the guidelines of the Declaration of Helsinki and approved by the Ethics Committee of the University of Medicine and Pharmacy of Craiova, Romania (No. 1481/30.05.2024) and sponsored by the Medicina Foundation.

## 3. Results

All continuous variables included in subsequent analyses with Student’s *t*-test were found to be normally distributed.

A total of 138 patients were included in the study. Of these, 75 (54.3%) were men and 63 (45.7%) were women. Baseline characteristics of the patients are presented in Table 1.

The mean age of the participants was 40.7 ± 10 years, with the majority (51.4%) belonging to the 31–40 years age group, followed by 20.3% (28 patients) in the 41–50 years group.

The parenterally infected patients belong to the Romanian pediatric cohort. They were during the 1986–1990 period and have been on ART for over two decades.

The prevalence of vitamin D deficiency (<20 ng/mL) and vitamin D insufficiency (20–29.9 ng/mL) was 36.2% and 33.3%, respectively, with an average vitamin D level of 26.4 ± 9.9 ng/mL.

According to BMI criteria, 5 patients (3.6%) were underweight, 58 (42%) patients had normal weight, 40 (29%) were overweight and 35 (25.4%) were classified as obese. We found that the mean BMI was 26.23 ± 19.17 kg/m^2^.

A significant association was found between body mass index categories and vitamin D deficiency (*p* = 0.0013). Individuals with higher BMI values, particularly those classified as overweight or obese, exhibited a greater prevalence of vitamin D deficiency compared to those with normal or low BMI (Table 2).

No significant association was found between vitamin D deficiency and demographic or lifestyle factors, including age, sex, smoking status, and alcohol consumption (all *p* > 0.05), but a significant association was observed between place of residence and vitamin D status. Individuals from urban areas showed a higher prevalence of vitamin D deficiency (50%) compared to those from rural areas (23.5%).

The CD4^+^ lymphocyte count, assessed prior to vitamin D measurement, had a median value of 816 cells/mm^3^ [29:1603] and the mean value was 634.2 ± 299.5 cells/mm^3^. The mean CD4^+^ lymphocyte nadir was 174.6 ± 152.8 cells/mm^3^.

Immunological assessment showed a median value of 647 cells/mm^3^ [min 125: max 1603] in patients with VDD and a median value of 750 cells/mm^3^ [min 29: max 1471] in patients with normal level of vitamin D. The mean CD4^+^ lymphocyte count was 648.54 ± 298 cells/mm^3^ in patients with VDD and 645.69 ± 300 cells/mm^3^ in patients with normal vitamin D level, with no statistically significant differences between these two groups (*p* = 0.957, Student’s *t*-test).

In total, 71 participants (51.4%) had a nadir CD4^+^ count below 200 cells/mm^3^, 34 (48%) having VDD, compared to 17 (33.3%, 17/51) who had nadir CD4^+^ ≥ 200 cells/mm^3^. There was a significant association between low nadir CD4^+^ count and VDD (*p* = 0.006, Chi-square test).

At the time of vitamin D measurement, 101 patients (73.2%) had an undetectable HIV viral load (<20 copies/mL). A greater number of patients with vitamin D deficiency had higher HIV viral loads (>10.000 copies/mL), compared to those without vitamin D deficiency (15.7% vs. 4.6%),with a mean of HIV viremia of 4.6 log10 copies/mL. Higher HIV viral load (>10.000 copies/mL) was significantly associated with VDD (*p* = 0.043, Chi-square test).

**Figure 1:**
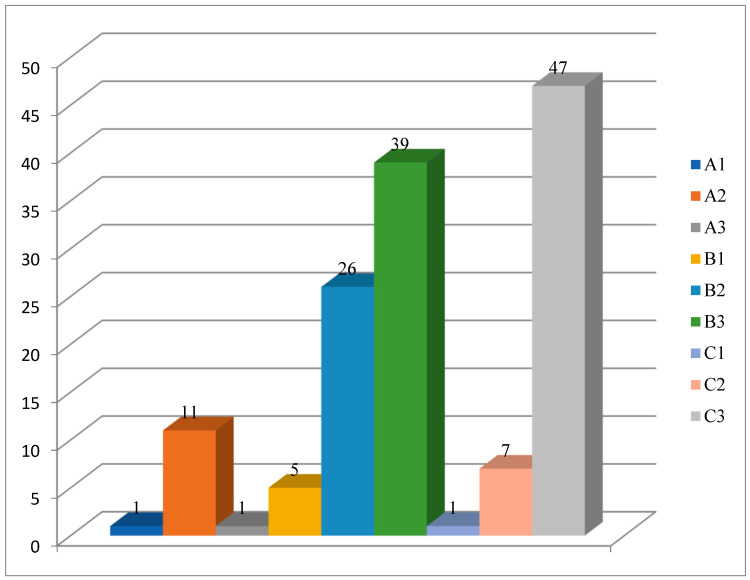
Stage of HIV infection.

The clinical and immunological classification of HIV infection, based on the CDC system, is illustrated in Figure 1. Overall, 68.8% of patients (95/138) were classified in the AIDS stage. Vitamin D deficiency was diagnosed in 33 patients (34.7%) within the AIDS group and in 18 patients (41.8%) in the non-AIDS group. No significant association was observed between HIV stage and vitamin D deficiency (*p* = 0.422, Chi-square test).

86 patients (62.3%) had been on ART for more than 15 years. The association between ART duration and vitamin D status was evaluated using the Chi-square test of independence. A statistically significant relationship was found (*p* = 0.002), indicating that patients receiving ART for more than 15 years had a higher prevalence of vitamin D deficiency compared to those treated for less than 15 years (Table 3).

An analysis of ART regimens and vitamin D status is presented in Table 4. Vitamin D deficiency was significantly associated with both TDF-containing and PI-containing antiretroviral regimens.

In the multivariate logistic regression model, several factors remained independently associated with vitamin D deficiency (Table 5). Overweight or obese individuals had a significantly higher likelihood of vitamin D deficiency compared to those with normal BMI (adjusted OR = 3.2, 95% CI: 1.6–6.4, *p* = 0.001). Urban residence was also independently associated with deficiency (adjusted OR = 3.0, 95% CI: 1.4–6.2, *p* = 0.003).

Patients with a CD4^+^ T-cell nadir below 200 cells/mm^3^ had increased odds of vitamin D deficiency (adjusted OR = 2.5, 95% CI: 1.3–5.0, *p* = 0.006). Similarly, a high HIV viral load (>10.000 copies/mL) was independently associated with deficiency (adjusted OR = 2.2, 95% CI: 1.0–4.8, *p* = 0.043).

Longer duration of ART (>15 years) was associated with a threefold increased risk of vitamin D deficiency (adjusted OR = 3.0, 95% CI: 1.5–6.2, *p* = 0.002). Additionally, exposure to TDF-containing regimens (adjusted OR = 2.1, 95% CI: 1.0–4.5, *p* = 0.034) and protease inhibitor-containing regimens (adjusted OR = 2.3, 95% CI: 1.2–4.7, *p* = 0.016) remained significant predictors after adjustment.

## 4. Discussion

PLWH commonly exhibit vitamin D deficiency, which has been associated with a wide range of conditions, including immune dysregulation and increased susceptibility to infections.

In this cohort from the Oltenia region of Romania, more than one third of patients exhibited vitamin D deficiency, while an additional third had vitamin D insufficiency, confirming that suboptimal vitamin D status is common even among individuals receiving long-term antiretroviral therapy. These findings are consistent with previous reports from diverse geographical settings, highlighting vitamin D deficiency as a persistent clinical issue in populations with HIV worldwide [23,24,25].

A significant association between increased BMI and vitamin D deficiency was observed in our study. Overweight and obese individuals showed a significantly higher likelihood of deficiency compared with patients with normal BMI. This relationship is biologically plausible, as vitamin D is a fat-soluble compound that becomes sequestered within adipose tissue, resulting in reduced circulating 25(OH)D concentrations and diminished bioavailability. In people living with HIV, this mechanism may be further exacerbated by ART-related metabolic changes, chronic low-grade inflammation, and reduced outdoor physical activity. Similar patterns have been observed in other studies, where higher BMI values were significantly correlated with lower 25(OH)D concentrations in individuals with HIV [26]. More studies involving both HIV-positive and HIV-negative populations indicated that obesity is an independent predictor of low vitamin D status [27,28].

In people living with HIV, this relationship may be further influenced by ART-related metabolic alterations, including changes in adipose tissue distribution and inflammatory adipokine profiles. Previous studies in multiexperienced HIV-treated patients have described associations between lipodystrophy, altered adipokine secretion (such as resistin), and metabolic disturbances, underscoring the complex interplay between long-term antiretroviral therapy, body composition, and metabolic regulation [29].

Given the growing prevalence of overweight and obesity among PLHIV as a consequence of effective ART and improved life expectancy, monitoring vitamin D status in this subgroup may have clinical importance.

The mean serum 25(OH)D level in our cohort was 26.4 ± 9.9 ng/mL, highlighting the widespread occurrence of suboptimal vitamin D levels among PLHIV. In a large cohort of 2044 patients with HIV, 89.2% had 25-hydroxyvitamin D [25(OH)D] levels < 30 ng/mL and 32.4% had levels < 10 ng/mL; the median value was 13.8 ng/mL (range 4–102). Low CD4 count (<200/µL) and efavirenz use were associated with the most severe deficiency [30].

In our cohort, vitamin D deficiency did not significantly differ by age, gender, smoking, or alcohol consumption. While some studies have reported associations between low vitamin D levels and older age, or female sex [30], our results may reflect the relatively young and homogeneous age distribution of our study population.

Urban residence emerged as an independent predictor of vitamin D deficiency in our cohort. This association likely reflects differences in lifestyle and environmental exposure, including reduced sunlight exposure, indoor occupational activities, and possibly higher levels of air pollution in urban settings. Previous studies have also documented lower vitamin D levels among urban residents compared with rural populations, suggesting that environmental and behavioral factors play a relevant role in vitamin D homeostasis. These findings highlight the need to consider place of residence as a contextual factor when assessing vitamin D status in individuals with HIV [30,31].

No significant differences were observed in current CD4^+^ counts between patients with or without vitamin D deficiency (mean 648.5 vs. 645.7 cells/mm^3^, *p* = 0.957). However, a low CD4^+^ nadir (<200 cells/mm^3^) was significantly associated with vitamin D deficiency (*p* = 0.006). This finding aligns with previous evidence suggesting that severe immunosuppression in the early stages of HIV infection may contribute to altered vitamin D metabolism and increased deficiency risk [3,28].

Previous longitudinal studies have reported associations between vitamin D deficiency and slower CD4^+^ T-cell count recovery among HIV-positive individuals initiating antiretroviral therapy [32]. These findings suggest a potential link between vitamin D status and immune reconstitution; however, causality cannot be inferred. Severe immunosuppression in early HIV infection may coexist with alterations in vitamin D metabolism, possibly reflecting shared underlying inflammatory and immunological mechanisms rather than a direct causal effect. Recent reviews have similarly described associations between vitamin D status and immunologic parameters in people living with HIV, while emphasizing the heterogeneity of available data and the uncertainty regarding clinical relevance [9,33]. Accordingly, while vitamin D has been proposed as a potentially modifiable factor, current evidence remains insufficient to determine whether correction of deficiency meaningfully influences immune recovery or HIV-related clinical outcomes. Further prospective studies and adequately powered randomized trials are required to clarify these relationships.

In our study, patients with higher HIV viral load (>10.000 copies/mL) had a significantly higher prevalence of vitamin D deficiency (*p* = 0.043), supporting the hypothesis that uncontrolled HIV replication may negatively impact vitamin D status.

The association between high HIV viral load and hypovitaminosis D likely reflects a bidirectional relationship: persistent viral replication induces chronic immune activation and inflammation, which can decrease vitamin D levels, while low vitamin D impairs T-cell function and immune recovery, potentially accelerating HIV disease progression.

Current data hint that vitamin D supplementation may improve immune outcomes in individuals with HIV, but additional research is required to establish evidence-based recommendations.

To explore whether correction of vitamin D deficiency may translate into immunological benefits, several intervention studies have evaluated vitamin D supplementation in people living with HIV. Randomized controlled trials using daily doses ranging from 1.600 to 4.000 IU of cholecalciferol administered for 6–12 months have consistently demonstrated effective increases in serum 25(OH)D concentrations, with modest effects on selected immune and inflammatory markers [18,34]. Higher-dose regimens, including daily supplementation of up to 7.000–10.000 IU or monthly loading doses, have been shown to be safe and capable of achieving sustained vitamin D sufficiency; however, their effects on CD4^+^ T-cell recovery and virologic outcomes have been variable [33,34]. While some studies report reductions in immune activation or inflammatory biomarkers, others fail to demonstrate significant improvements in clinical or virologic endpoints, underscoring the heterogeneity of supplementation protocols, study populations, and outcome measures [34,35]. Overall, current evidence supports the biochemical efficacy and safety of vitamin D supplementation in people living with HIV, but its impact on clinically meaningful immune outcomes remains incompletely established.

Vitamin D deficiency in our study population is likely multifactorial, with chronic immune activation and elevated inflammatory cytokines in HIV infection acting as primary drivers. These inflammatory processes may independently reduce vitamin D bioavailability and alter hydroxylation pathways, contributing significantly to hypovitaminosis D [1,36].

In our study, both the duration and type of ART were associated with vitamin D deficiency. Patients on ART for more than 20 years had a higher prevalence of deficiency (*p* = 0.002), likely reflecting cumulative effects on vitamin D metabolism. The use of protease inhibitors was significantly associated with vitamin D deficiency (*p* = 0.016). Similarly, patients receiving TDF-containing regimens were more likely to have a higher prevalence of vitamin D deficiency (*p* = 0.034).

Specific ART classes contribute: NNRTIs, such as efavirenz, have been linked to declines in serum 25(OH)D [13,37], while PIs, including ritonavir-boosted regimens, may impair the conversion of 25(OH)D to the biologically active 1,25(OH)_2_D [38].

Similar findings have been reported in cross-sectional studies. In Bangkok (2020–2021), individuals with HIV on ART had a higher prevalence of vitamin D deficiency compared with the general population [26]. In a Brazilian study of 125 adults with HIV, 24% were vitamin D deficient, with ART, obesity, female sex, sunscreen use, and history of opportunistic infections identified as significant risk factors [29].

Another cross-sectional study evaluated 125 adults with HIV in Brazil to identify risk factors associated with vitamin D deficiency. The prevalence of hypovitaminosis D was 24%, and ART was identified as a significant risk factor, along with obesity, female sex, sunscreen use, and history of opportunistic infections. These findings highlight that prolonged antiretroviral therapy may contribute to reduced vitamin D levels, with potential implications for immune function and bone health in people living with HIV [39].

Several studies have demonstrated an approximately 1–3% greater bone mineral density loss with TDF compared with other agents. Recent studies with tenofovir alafenamide have shown improved bone (and renal) safety with similar virologic efficacy when compared to TDF [40].

Taken together, the findings of this study suggest that vitamin D deficiency in people living with HIV is associated with a complex interaction of metabolic, immunological, virological, and treatment-related factors rather than demographic characteristics alone. The identification of potentially modifiable factors—such as body mass index, duration of antiretroviral therapy, and specific ART regimens—may help to better characterize subgroups of patients at higher risk for low vitamin D levels. While these observations do not support clinical recommendations, they highlight the need for further investigation into the role of vitamin D monitoring and supplementation within comprehensive HIV care models, particularly in patients with advanced immunosuppression or prolonged ART exposure.

These findings underscore the potential value of routine vitamin D monitoring in PLHIV, particularly among those with higher-risk profiles, such as severe immunosuppression, elevated viral load, long-term ART exposure, or regimens containing TDF or PIs. Early identification of deficiency and appropriate supplementation could help prevent bone-related complications and may support immune function, thereby potentially contributing to better overall health outcomes in this population.

This study has several limitations. The small sample size (138 patients) was largely due to the exclusion criteria, particularly patients with comorbidities affecting vitamin D metabolism and women with osteoporosis who were already receiving vitamin D supplementation. The retrospective, single-center design limits generalizability and precludes causal or prognostic inference. Reliance on existing medical records restricted the availability of standardized biochemical and clinical data for all participants. Vitamin D status was assessed at a single time point, preventing evaluation of temporal trends or longitudinal associations. Additionally, important confounders—such as dietary vitamin D intake, sunlight exposure, seasonal variation, physical activity, and ART adherence—were not assessed and may have influenced the observed associations.

## 5. Conclusions

In this retrospective observational study of PLHIV registered at the CRC, we observed a high prevalence of vitamin D deficiency, with 36.2% of patients exhibiting deficiency (<20 ng/mL) and an additional 33.3% showing insufficiency.

Lower nadir CD4^+^ cell counts (<200 cells/mm^3^) and higher HIV viral load (>10.000 copies/mL) were more common among patients with vitamin D deficiency, reflecting an association with markers of immunological status and viral replication in our cohort.

Overweight and obese patients more frequently exhibited lower vitamin D levels, a pattern consistent with previous reports in the literature showing an association between higher BMI and lower serum vitamin D concentrations.

Furthermore, longer duration of ART (>15 years) and exposure to regimens containing TDF or PIs were more frequently observed among patients with lower vitamin D levels, consistent with patterns previously described in PLHIV and may merit further study.

Given the small sample size, these findings should be considered preliminary and hypothesis-generating, warranting confirmation in larger cohorts.

## Figures and Tables

**Table 1:** Baseline characteristics of patients with HIV.

Characteristics		Number of Patients (%)
Patients’ age (years old)	21–30	11 (8%)
	31–40	71 (51.4%)
	41–50	28 (20.3%)
	51–60	21 (15.2%)
	>60	7 (5.1%)
Body mass index (kg/m^2^)	>18.5	5 (3.6%)
	18.5–24.9	58 (42%)
	25–29.9	40 (29%)
	≥30	35 (25.4%)
Probable route of HIV transmission	Parenteral	53 (38.4%)
	Sexual	83 (60.1%)
	Mother-to-child	2 (1.5%)
Smoking	Yes	59 (42.8%)
	No	79 (57.2%)
Alcohol consumption	Yes	50 (36.2%)
	No	88 (63.8%)
Place of residence	Rural	68 (49.3%)
	Urban	70 (50.7%)

**Table 2:** Demographic data.

Characteristics		Vitamin D < 20 ng/mL (*n* = 51)	Vitamin D > 20 ng/mL (*n* = 87)	*p* Value
Patients’ age (years old)		39.49 ± 10.08	41.34 ± 9.94	0.295 (Student’s *t*-test)
Gender	Male	24 (47.1%)	51 (58.6%)	0.480 (Fisher’s test)
	Female	27 (52.9%)	36 (41.4%)	
BMI (kg/m^2^)	Underweight	1 (2%)	4 (4.6%)	0.0013 (Chi-square test)
	Normal weight	12 (23.5%)	46 (52.9%)	
	Overweight	17 (33.3%)	23 (26.4%)	
	Obesity	21 (41.2%)	14 (16.1%)	
Place of residence	Urban	35 (68.6%)	35 (40.2%)	0.0013 (Chi-square test)
	Rural	16 (31.4%)	52 (59.8%)	
Smoking	Yes	25 (49%)	34 (39.1%)	0.287 (Fisher’s test)
	No	26 (51%)	53 (60.9%)	
Alcohol consumption	Yes	20 (39.2%)	30 (34.5%)	0.587 (Fisher’s test)
	No	31 (60.8%)	57 (65.5%)	
CD4 count (cells/mm^3^)	<200	3 (5.9%)	4 (4.6%)	0.853 (Chi-square test)
	200–500	11 (21.6%)	22 (25.3%)	
	>500	37 (72.5%)	61 (70.1%)	

**Table 3:** ART duration and VDD.

ART Duration	Vitamin D Deficiency, *n* (%)	Normal Vitamin D, *n* (%)	Total, *n* (%)
>15 years	40 (46.5%)	46 (53.5%)	86 (100%)
≤15 years	11 (21.2%)	41 (78.8%)	52 (100%)
Total	51 (36.7%)	87 (63.3%)	138 (100%)

**Table 4:** Association between ART regimens and VDD.

ART Regimen	Vitamin D < 20 ng/mL *n* (%)	Vitamin D > 20 ng/mL *n* (%)	*p* Value
TDF-containing regimens (*n* = 65)	30 (46.2%)	35 (53.8%)	0.034 (Chi-square test)
PIs-containing regimens (*n* = 62)	29 (46.8%)	33 (53.2%)	0.016 (Chi-square test)

**Table 5:** Multivariate logistic regression analysis of factors associated with VDD in PLWH.

Variable	Adjusted OR	95% CI	*p*-Value
Overweight/obese (vs. normal BMI)	3.2	1.6–6.4	0.001
Urban residence (vs. rural)	3.0	1.4–6.2	0.003
CD4^+^ T-cell nadir < 200 cells/mm^3^	2.5	1.3–5.0	0.006
HIV viral load > 10.000 copies/mL	2.2	1.0–4.8	0.043
ART duration > 15 years	3.0	1.5–6.2	0.002
TDF-containing regimen	2.1	1.0–4.5	0.034
PI-containing regimen	2.3	1.2–4.7	0.016

## Data Availability

Data is contained within the article. Further inquiries can be directed to the corresponding authors.
